# Discrepancies between UN models and DHS survey estimates of maternal orphan prevalence: insights from analyses of survey data from Zimbabwe

**DOI:** 10.1136/sti.2008.029926

**Published:** 2008-07-22

**Authors:** L Robertson, S Gregson, C Madanhire, N Walker, P Mushati, G Garnett, C Nyamukapa

**Affiliations:** 1 Department of Infectious Disease Epidemiology, Imperial College, London, UK; 2 Biomedical Research and Training Institute, Harare, Zimbabwe; 3 Department of International Health, Johns Hopkins Bloomberg School of Public Health, Baltimore, Maryland, USA

## Abstract

**Objectives:**

Model-based estimates of maternal (but not paternal) orphanhood are higher than those based on data from demographic and health surveys (DHS). We investigate the consistency of reporting of parental survival status in data from Manicaland, Zimbabwe.

**Methods:**

We compared estimates of paternal and maternal orphan prevalence in three rounds of a prospective household census in Manicaland (1998–2005) with estimates from DHS surveys and UNAIDS model projections. We investigated the consistency of reporting of parental survival status across the three rounds and compared estimates of adult mortality from the orphan data with direct estimates from concurrent follow-up of a general population cohort. Qualitative data were collected on possible reasons for misreporting.

**Results:**

Paternal and maternal orphan prevalence is increasing in Zimbabwe. Mothers reported as deceased in round 1 of the Manicaland survey were more likely than fathers to be reported as alive in rounds 2 or 3 (33.3% vs 13.4%). This pattern was most apparent among younger children. The qualitative findings suggest that foster parents sometimes claim adopted children as their natural children.

**Conclusions:**

These results are consistent with misreporting of foster parents as natural parents. This appears to be particularly common among foster mothers and could partly explain the discrepancy between mathematical model and DHS estimates of maternal orphanhood.

Direct empirical estimation of orphan numbers in many developing countries comes from demographic and health surveys (DHS). These are national, cross-sectional, household surveys conducted every 4–5 years. Concomitantly, UNAIDS, UNICEF, USAID and the US Census Bureau have developed mathematical models (Spectrum)^1^ to produce demographic projections of maternal, paternal and dual orphan prevalence—both all-cause and orphans as a result of AIDS—in countries experiencing major HIV epidemics, based on trends in HIV prevalence.^2^^–^5

A comparison of the 2001 estimates of all-cause orphans found that maternal orphan estimates from Spectrum were significantly higher than those in DHS.^6^ This difference was unrelated to national HIV prevalence among countries in sub-Saharan Africa, suggesting that the differences were not caused by assumptions about AIDS deaths. The assumptions about underlying adult and child mortality—Spectrum uses the United Nations Population Division’s population projections—could have caused an overestimate of maternal orphans. Alternatively, the household surveys methodology could have led to a systematic bias in the reporting of maternal orphans.

UNAIDS and its technical partners used updated UN Population Division model life tables to produce revised estimates of orphans.^7^ However, these new mathematical models still overestimate the prevalence of maternal orphanhood when compared to the DHS estimates across several sub-Saharan Africa countries (fig 1), although the difference is less pronounced than for the 2001 estimates.^8^ The model predictions of the prevalence of paternal orphanhood compare well with the DHS estimates.

**Figure 1:**
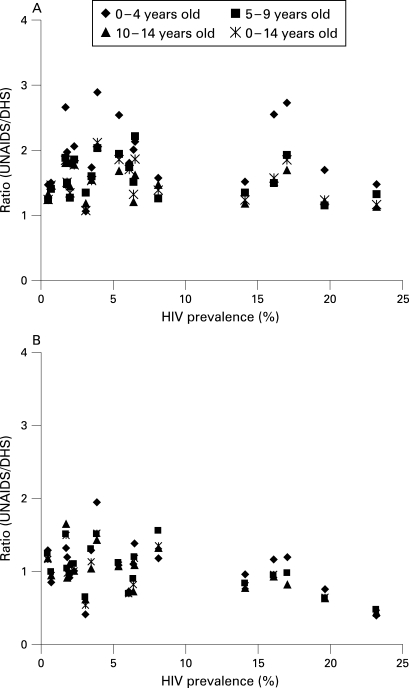
(A) Ratio of Spectrum estimates of maternal orphans over DHS estimates, by age and adult HIV prevalence. (B) Ratio of Spectrum estimates of paternal orphans over DHS estimates, by age and adult HIV prevalence.

Orphan data are also used to indirectly estimate adult mortality in countries without vital registration systems.^9^^–^12 This method can give biased estimates if there is under-reporting of children whose parents died when they were young, because of reports being based on the survival status of their living foster parents—the “adoption effect”.^11^ ^12^ If foster mothers are more likely than foster fathers to be misreported as natural parents, this could partly explain the discrepancy between the model and DHS estimates of maternal orphan prevalence.

We use data from an open cohort study in Manicaland, Zimbabwe, to investigate bias in the reporting of maternal and paternal orphanhood. Qualitative methods were used to explore possible reasons for the biases found.

## METHODS

### Demographic and health surveys

Demographic and health surveys (DHS) are national, cross-sectional, household surveys. In each survey, a sample ranging from 3500 to 9000 households is selected in each country using a stratified random sample of clusters that are chosen to be representative of urban and rural areas. DHS have been conducted in Zimbabwe in 1988, 1994, 1999 and 2005-6.^13^^–^16 A questionnaire is completed for each household in which all individuals resident in the household are listed. For each child under 18 years (2005-6) or under 15 years (1988–99) the question “Is NAME’s biological mother/father alive?” is asked. A child was defined as a paternal orphan if their father had died regardless of the survival status of their mother—that is, the definition of paternal orphans includes double orphans. A similar definition for maternal orphans was used. Prevalence of orphanhood among children aged 0–14 years was calculated in Manicaland for each round of the DHS (excluding 1988 for which the data could not be accessed). The prevalence of missing and unknown orphan status was also calculated.

### Manicaland survey—quantitative data

An open cohort study looking at the demographic impact of HIV/AIDS is currently under way in Manicaland, a rural province in eastern Zimbabwe. Three survey rounds have been completed—July 1998 to February 2000, July 2001 to February 2003 and July 2003 to August 2005.^17^ The surveys cover four socioeconomic strata—subsistence farming areas, roadside trading settlements, agricultural estates and commercial centres. A census of the households in each area is undertaken at each round. An adult from each household is asked “Is NAME’s natural mother/father still alive?” for all resident children aged under 16 years (round 1 and round 2) or 18 years (round 3). The year of death of the parents is also collected and the identity of the parent is checked on birth certificates whenever possible. In the second and third survey rounds, the responses from the previous round were pre-printed on the questionnaires in an attempt to improve consistency. In round 3, individuals aged 15–54 years were eligible to complete a detailed individual questionnaire.

More detailed information about the orphan status of each participant is available in the Manicaland data than the DHS and since the study follows the same individuals repeatedly over time, it is possible to validate responses by cross-checking with answers given in other rounds.

Prevalence of missing and unknown parental survival status was calculated at each round of the survey for children aged 0–14 years. For those children with complete data, the prevalence of each type of orphanhood was then calculated. The remaining analysis used only the children aged 16 years or less at last birthday who were reported as maternal, paternal or double orphans at round 1 and were followed up in the second and third rounds of the study. The proportions of these children whose mothers and fathers were reported as deceased at round 1 but as alive at rounds 2 or 3 were compared. To minimise data processing errors, all inconsistencies were checked against the original questionnaires and corrected when necessary. The relation between inconsistency of reporting and the sex, age and double orphan status of the child, poverty quintile and whether the birth certificate had been checked was investigated.

During the household census, the orphan status of each child is reported by whichever resident adult is available to complete the survey. Children aged 15–17 years were eligible to complete an individual questionnaire, which repeats the parental survival questions. This self-reported status was considered as a “gold standard” and compared to the household census data from round 3 for children aged 15–17 years. The sensitivity and specificity of household census reports of orphan status were calculated based on this comparison.

The data on parental survival for children aged 5–14 years at round 3 were also used to calculate indirect estimates of adult mortality using the orphanhood method.^9^ ^10^ ^18^ ^19^ Attempts were made to use correction factors that adjust for the effects of different age-patterns of adult mortality in populations experiencing large HIV epidemics and for the bias introduced by children of HIV-positive mothers being selected out of the population—because of vertical transmission of HIV and reduced fertility among HIV-positive women.^20^ The estimates were then time located^19^ ^21^ and compared to direct estimates of adult mortality based on data collected on the survival of adults aged 15–49 years between successive rounds of the Manicaland survey. The probability of dying between their 15th and 50th birthdays (_35_q_15_) was then calculated using each method.

Ethical approval for the Manicaland study was obtained from the Biomedical Research and Training Institute’s institutional review board (Number AP6/97), the Research Council of Zimbabwe (Number 02187) and the Applied and Qualitative Research ethics committee in Oxford, United Kingdom (M97.039).

### Manicaland—qualitative data

A focus group discussion was held in Bonda, a subsistence farming area and study site in the Manicaland survey. Nine female respondents with children living in their households were recruited. Discussions were conducted in Shona, recorded by hand and translated into English by a researcher (CM). Discussions were held on two topics: (1) reasons for the inconsistent reporting of parental deaths; and (2) the process of birth registration following a parental death. The discussion lasted approximately 1 hour.

A fourth round of the Manicaland survey is currently under way. Key informant interviews were conducted in four households in Bonda where parents reported as deceased in round 3 were then reported as alive in round 4. Attempts were made to ascertain the reasons for the discrepancy and also to discuss more generally why such discrepancies might occur. The interviews were conducted in Shona, recorded by hand and translated into English by a researcher (CM).

## RESULTS

### Quantitative findings

The results in table 1 show that the prevalence of all types of orphanhood has been increasing in Zimbabwe. There is greater prevalence of paternal orphans than maternal orphans at all time points. The prevalence of each type of orphanhood is lower in the Manicaland cohort study compared to DHS Manicaland in 1999 and 2003-6 (table 1). This difference may be the result of the smaller sample size of the DHS Manicaland compared with the Manicaland cohort study. Moreover, the DHS sample frame is designed to produce a representative estimate for the whole of Zimbabwe, whereas the Manicaland cohort study collects data from specifically defined socioeconomic strata, which may not be representative of the area overall. Reports of unknown (but not missing) survival status were more common for fathers than for mothers in the Manicaland cohort study and DHS Manicaland (table 1). Levels of missing and unknown survival status increased in successive rounds of the Manicaland census for both mothers and fathers. The DHS had low prevalence of missing and unknown data for both maternal and paternal survival status at all time points.

**Table 1:** Prevalence of orphanhood and missing or unknown parental survival status among children aged 0–14 years from Zimbabwe

	DHS Manicaland 1994		DHS Manicaland 1999		Manicaland cohort study 1998–2000		Manicaland cohort study 2001–3		DHS Manicaland 2005–6		Manicaland cohort study 2003–5	
	%	No	%	No	%	No	%	No	%	No	%	No
Orphan prevalence*												
Maternal orphans	3.1	1342	5.8	1298	3.2	13 338	6.2	7712	10.3	2377	7.7	13 644
Paternal orphans	10.6	1312	14.0	1253	10.9	13 267	16.7	7604	23.1	2339	18.0	13 344
Double orphans	1.4	1312	2.9	1252	1.4	13 220	3.1	7568	7.1	2336	4.3	13 310
All types	12.1	1312	16.9	1252	12.5	13 220	19.4	7568	25.9	2336	20.8	13 310
Missing parental survival status												
Maternal	1.5	1363	0.2	1301	2.1	13 628	6.2	8233	0.1	2382	9.7	15 142
Paternal	1.8	1363	0.5	1301	1.8	13 628	5.9	8233	0.1	2382	9.7	15 142
Unknown parental survival status												
Maternal	0.0	1363	0.1	1301	0.1	13 628	0.1	8233	0.1	2382	0.2	15 142
Paternal	1.9	1363	3.2	1301	0.8	13 628	1.7	8233	1.8	2382	2.2	15 142

1988 DHS not included as the data were unavailable.

*Numbers include children with non-missing parental survival data only**.**

In total, 11 984 children under 16 years at round 1 were followed up in rounds 2 and 3 of the Manicaland study. Among the 198 children reported as maternal orphans in the first round, 66 (33.3%) reported a living mother in either round 2 or round 3 (table 2). Of the 689 children reported as paternal orphans in the first round, 92 (13.4%) reported a living father at either round 2 or round 3. Mothers who were reported as deceased in round 1 and alive in round 2 were more likely than fathers in the same situation to continue to be reported as alive in round 3 (50.0% vs 30.4%; table 2).

**Table 2:** Inconsistency of reporting of orphan status in three rounds of census data from Manicaland, Zimbabwe (1998–2005)

	Maternal orphans at R1 (n = 198)		Paternal orphans at R1 (n = 689)	
	No (%)	95% CI	No (%)	95% CI
Non-orphans at R2 or R3	66 (33.3)	26.7 to 39.9	92 (13.4)	10.9 to 15.9
Pattern of inconsistency over three rounds				
Orphan–non-orphan–orphan	21 (31.8)	20.6 to 43.0	47 (51.1)	40.9 to 61.3
Orphan–orphan–non-orphan	12 (18.2)	8.9 to 27.5	17 (18.5)	10.6 to 26.4
Orphan–non-orphan–non-orphan	33 (50.0)	37.9 to 62.1	28 (30.4)	21.0 to 39.8

Table 3 shows the associations between various sociodemographic variables and consistency of reporting of parental survival. Younger children were more likely than older children to report a parent as deceased at round 1 but alive in later rounds. This was true for both mothers and fathers (χ^2^ test for trend: maternal p = 0.004; paternal p = 0.031; not shown in table 3). Being reported as a double orphan increased the likelihood of deceased fathers being reported alive in later rounds (double orphans 24.7% vs single paternal orphans 11.9%; p = 0.002) but the same was not true for deceased mothers (double orphans 32.1% vs single maternal orphans 35.4%; p = 0.631). Inconsistency with reporting in subsequent rounds was highest in agricultural estates for children reported as maternal orphans (48.9% of deceased mothers later reported as alive) and paternal orphans (19.4% of deceased fathers later reported as alive) at round 1. Least inconsistency was found for paternal orphans in commercial centres (5.6% of deceased fathers later reported alive) and for maternal orphans in subsistence farming areas (22.3% of deceased mothers later reported alive).

**Table 3:** Determinants of parents reported as deceased at round 1 being reported as alive at a later round, Manicaland census data (1998–2005)

Determinants	Maternal orphans				Paternal orphans			
	%	No	OR (95% CI)	p Value	%	No	OR (95% CI)	p Value
Type of orphan								
Not a double orphan	35.4	113	1	0.631	11.9	607	1	0.002
Double orphan	32.1	78	0.86 (0.47 to 1.59)		24.7	73	2.43 (1.35 to 4.39)	
Sex of child								
Male child	34.6	110	1	0.686	13.8	347	1	0.709
Female child	31.8	88	0.88 (0.47 to 1.61)		12.9	342	0.92 (0.59 to 1.43)	
Age of child								
0–3 years	47.6	21	1	0.025	19.0	79	1	0.174
4–7 years	42.2	19	0.80 (0.28 to 2.30)		14.7	191	0.73 (0.37 to 1.47)	
8–11 years	32.7	33	0.53 (0.20 to 1.40)		12.8	329	0.62 (0.33 to 1.20)	
12–15 years	12.9	4	0.16 (0.04 to 0.71)		7.8	90	0.36 (0.14 to 0.95)	
Poverty quintile								
1st (poorest)	15.6	32	1	0.134	11.1	135	1	0.736
2nd	41.2	34	3.78 (1.10 to 12.97)		11.9	126	1.08 (0.50 to 2.32)	
3rd	28.6	56	2.16 (0.69 to 6.72)		15.3	176	1.45 (0.74 to 2.86)	
4th	40.5	37	3.68 (1.10 to 12.38)		15.0	127	1.41 (0.68 to 2.91)	
5th (richest)	36.1	36	3.05 (0.91 to 10.27)		11.7	120	1.06 (0.49 to 2.29)	
Type of location								
Subsistence farming area	22.3	94	1	0.009	14.1	320	1	0.030
Roadside trading settlement	41.5	41	2.46 (1.10 to 5.52)		10.7	168	0.73 (0.41 to 1.31)	
Agricultural estate	48.9	47	3.33 (1.52 to 7.28)		19.4	129	1.47 (0.86 to 2.52)	
Commercial centre	31.3	16	1.58 (0.49 to 5.10)		5.6	72	0.36 (0.12 to 1.04)	
Checked birth certificate								
All three rounds	31.1	35	1	0.787	10.3	156	1	0.302
At least one round	36.5	104	0.97 (0.44 to 2.16)		14.3	435	1.45 (0.81 to 2.61)	
No rounds	30.3	33	0.74 (0.27 to 2.04)		13.2	68	1.34 (0.56 to 3.12)	

Among children aged 16 years or less at round 1, 57%, 49% and 46% had their birth certificates checked at rounds 1, 2 and 3, respectively. Among children aged 16 years or less who were followed up in all three rounds, 11% had their birth certificates checked at every round. Checking the birth certificate at all three rounds did not significantly reduce the probability of children reported as orphans in round 1 being reported as non-orphans in later rounds for either maternal orphans (37.1% among those checked at all rounds vs 30.3% among those never checked; p = 0.787) or paternal orphans (10.3% vs 13.2%; p = 0.449).

Among children aged 15–17 years, the sensitivity of household census reports versus self-reports was 93.7% (355/379) for maternal orphan status and 92.7% (689/743) for paternal orphan status. The specificity was 98.6% (1850/1877) for maternal orphan status and 98.1% (1414/1442) for paternal orphan status.

Using data on maternal survival from 5–9 and 10–14-year-olds and applying the correction factor for selection of children whose mothers are still alive, the probability of a woman dying between her 15th and 50th birthdays (_35_q_15_) was found to be greater than 0.50 (the largest value on the model life tables used). When the correction factor was not applied, the corresponding _35_q_15_ values were 0.37 and 0.28, respectively. These sets of estimates were time-located to 2001 for the estimates based on the 5–9 year-olds and 1999 for the estimates based on the 10–14 year-olds. Thus the most appropriate direct comparison is with _35_q_15 _estimated from data on adult deaths occurring between round 1 (1998–2000) and round 2 (2001–3) of the Manicaland adult cohort survey, which, for women, was 0.36. The _35_q_15_ for men based on reported paternal survival of children aged 5–14 years, without corrections for selection bias or differences in the age patterns of adult mortality in populations experiencing large HIV epidemics, was 0.43. This was time located to 1999 and was compared to a direct estimate of 0.53 based on adult deaths occurring between rounds 1 and 2 of the Manicaland cohort survey.

### Qualitative findings

The focus group consisted of nine women from the community whose ages ranged from 30–56 years. Four were married, two were divorced and three were widowed. Five had received secondary school education, three had attended primary school and one had received no education.

The most common reasons given for the misreporting of orphan status were that the respondents had lied, that they had not understood the questions or that they were frightened to reveal the truth—the inconsistency would arise when someone forgot their previous response.

“People are just not free to let out everything about their families. They don’t want to tell the truth about their families to strangers so people might even lie because they are not sure about how this household information would be used.” *Married woman aged 54 years*

“Some people misrepresent the truth out of the expectation of getting something. For example, some people claim to be living with orphans with the hope of getting material assistance.” *Divorced woman aged 38 years*

Some respondents thought it was possible that foster parents might claim adopted children as their own natural children, especially if they were related to the child. Others thought this was unlikely to happen. It was stated that foster mothers would be more likely to do this than foster fathers, although no explanation for this was provided. It was suggested that the inconsistency in responses would be more common among older children. For paternal orphans, fathers might refuse responsibility for a child when he/she is young—and therefore may be recorded as dead—but might claim the child when he/she is older.

“Some fathers never accept responsibility for their children so they might not even bother to find where their children are living. So such children’s fathers might be reported as dead, but these fathers would only come to claim their children when they are a bit older.” *Married woman aged 54 years*

The group thought it was possible for foster parents to be listed as natural parents on the birth certificate, especially if the foster parents were related to the natural parents, but that the children themselves would always be told the truth (although some may feel ashamed of their orphan status).

“It is only possible when our daughters give birth out of unwanted pregnancy and the boy refuses responsibility. When the daughter gets married later on to someone else, the child would live with its grandparents and the grandmother may claim to be its mother and go to get the birth certificate for the child as her own.” *Married woman aged 52 years*

Four key informant interviews were conducted in households where parents had been reported as dead in round 3 but alive in round 4. The reasons given for these inconsistencies were either that the researcher conducting the interview had made a mistake (3/4) or that another member of the household (though not the child who’s parents’ survival status was inconsistently reported) had answered the questions wrongly (1/4).

## DISCUSSION

The prevalence of all types of orphanhood has risen substantially in Zimbabwe but, as in several other countries, UNAIDS Spectrum-based estimates of maternal loss are higher than empirical survey estimates. In a closed cohort of children reported as orphans in the first of three household censuses in Manicaland, eastern Zimbabwe, where comparable increases in orphanhood have been observed, consistency of reporting of orphan status was lower for maternal orphans than for paternal orphans—33.3% of maternal orphans in the first round of the study were reported as having a living mother in either round 2 or round 3 compared to 13.4% of paternal orphans. Inconsistent reporting was more common for younger children than for older children for both maternal and paternal orphanhood. Mothers reported as deceased in round 1 and alive in round 2 were more likely than fathers in the same situation to continue to be reported as alive in round 3. Comparisons of reported orphan status for older children (15–17 years) in household census questionnaires with a gold-standard of self-reported orphan status showed high levels of sensitivity and specificity for both maternal and paternal orphans.

These findings are consistent with the possibility that selective under-reporting of maternal mortality in household surveys contributes to the lower levels of maternal orphanhood seen in surveys (particularly at young ages) when compared to model estimates. Checking birth certificates did not significantly improve the consistency of reports but other procedures followed in the Manicaland censuses such as the recording of parents’ years of death on the household forms would be expected to have improved reliability. Our analysis is limited as we only detect individuals reported as orphans whose parents are subsequently reported as alive. Other potential inconsistencies could not be measured. We may have underestimated the full extent of underenumeration of orphans because children incorrectly reported as non-orphans at round 1 were not identified subsequently. However, in the opposite direction, children whose parents were still alive could have been incorrectly reported as orphans in the censuses. If this occurred in the first round and was then corrected at later rounds, it would lead us to believe that under-reporting of orphans was greater than was really the case.

Data on parental survival have been used to estimate adult mortality in countries with incomplete vital registration. Some analyses have found mortality estimates based on orphan data to be consistent with other estimates of mortality.^10^ ^22^^–^24 However, many other studies have found that this method underestimates adult mortality^11^ ^25^^–^29 and it has been suggested that this could be due to biases in the data introduced by the adoption effect—that is, children whose parents have died may later be adopted by foster parents who are subsequently incorrectly reported as being the natural parents.^11^ ^12^ No direct comparisons have been made of this bias for estimates of female versus male mortality. However, it has been found that the bias is greater among younger respondents,^6^ which is consistent with the findings here. In the current study, an indirect estimate of female adult mortality (_35_q_15_) between 1998 and 2000 of >0.50 was obtained from the reported orphanhood data for 5–14-year-olds. This was higher than the corresponding direct estimate of 0.36 obtained from follow-up of a population cohort. However, migration is common in this predominantly rural population^30^ and children with parents who lived in urban and other centres of employment are frequently relocated to rural areas when their parents become sick or die.^31^^–^33 This bias may over-ride that due to under-reporting of orphanhood. In addition, the direct estimates of adult mortality could also be underascertained if adults who die are selectively lost to follow-up.

The pattern of inconsistency in reporting observed in our data may be explained by the adoption effect. If this was more common among foster mothers than foster fathers, it could explain why the consistency in reporting of maternal survival is lower than that for paternal survival. This is supported, to a degree, by the finding that children whose mothers were reported as deceased in round 1 and alive in round 2 were more likely to continue to be reported as alive in round 3 than was the case for those whose fathers were initially reported as deceased. Also, respondents in the small qualitative study agreed that foster mothers, in particular, might claim to be the natural parents, especially if they were related to the child. Paternal survival status was more likely to be unknown than maternal survival status in the Manicaland censuses and DHS. Thus, inconsistencies in reporting of fathers’ survival status could be largely the result of uncertainty over the identity and/or current whereabouts of the father, whereas reporting of mothers’ survival status may be subject to a systematic bias due to the adoption effect. The increase in unknown and missing data across successive rounds of Manicaland censuses may be the result of increased mobility in recent years leading to increased uncertainty about the survival status of household members. It is also possible that pre-printed information from previous rounds, which does not change in later rounds, is not being entered into the database by the research assistants as new members (with no pre-printed information) were less likely to have missing data than old members (data not shown).

Blacker and Mukiza-Gapere^26^ attempted to reconcile inconsistencies in estimates of female mortality based on orphan data from Kenya by adjusting for the possible bias introduced by the adoption effect. They found that, in the 5–14-year age range, a third of children whose mothers were deceased would need to be reported as alive in order to reconcile the data. However, they also suggested that this represented a maximum value since other factors would also contribute to bias in the estimates.

Further studies are needed to measure levels of underestimation of maternal orphanhood in survey data in populations subject to HIV epidemics of different magnitudes and at different stages. Such measurements can inform assessment of the contribution of this bias to the discrepancy between Spectrum projections and national survey estimates and could provide the basis for developing correction factors for orphan estimates derived from survey data. Our findings also suggest that improved consistency in the collection of parental survival data in household surveys is required. In particular, steps should be taken to minimise the misreporting of foster parents as natural parents. Additionally, our qualitative investigations highlighted other possible sources of bias in orphanhood data—for example, shame about reporting orphan status and misunderstanding of parental survival questions. Efforts should be made to reduce the impact of these biases when developing interview protocols for use in future household surveys.

**Key messages**

- Model-based estimates of maternal (but not paternal) orphanhood are higher than those based on data from demographic and health surveys (DHS) across sub-Saharan Africa.
- Mothers reported as deceased in round 1 of a prospective household census in eastern Zimbabwe were more likely than fathers to be reported as alive in rounds 2 or 3.
- Qualitative research suggests that the misreporting of foster mothers as natural mothers is possible in household surveys.
- This misreporting could lead to an underestimate of maternal orphans in household surveys, which may explain the discrepancy between the model and DHS estimates of maternal orphan prevalence.
